# What Is a Blush?

**Published:** 1894-07

**Authors:** 


WHAT IS A BLUSH?—It seems that, unlike an osculatory demonstration, a blush can be scientifically defined. A Cincinnati physician attempts it as follows:

"A blush is a temporary erythema and calorific effulgence of the physiognomy, etiologzied by the perceptiveness of the sensorium when in a predicament of unequilibrity from a sense of shame, anger, or other cause, eventuating in a paresis of the vaso-motor nervous filaments of the facial capillaries, whereby, being divested of their elasticity, they are suffused with a radiance emanating from an intimidated præcordia."—*Ex.*

